# Effects of Mineral Raw Materials on Melting–Crystallization Properties and Microstructure of Fluorine-Free Mold Flux for High-Titanium Steel Continuous Casting

**DOI:** 10.3390/ma19122600

**Published:** 2026-06-17

**Authors:** Di Zhang, Xiuli Han, Lei Liu, Ziyao Liu, Yue Yang, Lei Wu, Ziyi Zhang

**Affiliations:** 1 College of Mining Engineering, North China University of Science and Technology, Tangshan 063210, China; 2 Collaborative Innovation Center of Green Development and Ecological Restoration of Mineral Resources, Tangshan 063210, China; 3 College of Metallurgy and Energy, North China University of Science and Technology, Tangshan 063210, China

**Keywords:** fluorine-free mold flux, mineral raw materials, melting properties, crystallization properties, microstructure, high-titanium steel

## Abstract

During the continuous casting of high-titanium steel, traditional fluorine-containing mold fluxes are prone to causing fluoride contamination, equipment corrosion, and intensified slag–metal interface reactions. There is an urgent need to develop highly adaptable fluorine-free mold flux systems. In this study, titanium-containing blast furnace slag was used as the primary base material, while borax, soda ash, and witherite were selected as fluoride-substituting mineral raw materials. The effects of these mineral raw materials on the melting properties, crystallization behavior, crystalline phases, and microstructure of fluorine-free mold fluxes were systematically investigated, and an optimized mold flux design suitable for continuous casting of high-titanium steel was further developed. The results indicate that borax significantly reduces the melting temperature and viscosity and markedly suppresses the growth of crystalline phases such as calcium borosilicate, nepheline, and perovskite by weakening the polymerization degree of the silicate network, thereby substantially decreasing the crystallization ability of the mold flux. Soda ash primarily acts as a strong fluxing and network-depolymerizing agent, promoting the formation of low-polymerized structural units. It also enhances the tendency toward ordered atomic arrangement, thereby markedly increasing nepheline precipitation and the overall crystallization ratio. Witherite exerts a relatively mild effect on slag structure and phase evolution; its moderate addition helps synergistically reduce the melting point, viscosity, and crystallization ratio, thereby supporting performance stability. The optimized fluorine-free mold flux, designed on the basis of these findings, maintains a suitable initial crystallization temperature and critical crystallization cooling rate while exhibiting lower melting temperature, viscosity, and crystallization ratio than conventional fluorine-bearing flux. The findings establish a theoretical basis for designing eco-friendly mold fluxes suitable for high-titanium steel and for enhancing billet quality.

## 1. Introduction

Due to their excellent comprehensive mechanical properties and service adaptability, high-titanium steels demonstrate significant application value in fields such as marine engineering, energy equipment, and high-end structural materials [1,2,3,4]. As the requirements for billet surface quality, internal uniformity, and production stability in special steels continue to rise, the performance compatibility of mold flux during the continuous casting process has become a critical factor influencing billet quality [5,6,7]. In fact, compared to ordinary steel grades, the continuous casting process for high-titanium steel imposes stricter requirements on mold flux. This is primarily because, when the properties of the mold flux are mismatched with the continuous casting process, the Ti in the molten steel is highly prone to violent interfacial reactions with the mold flux. This leads to insufficient lubrication, uneven heat flux distribution, and instability in the growth of the slab skin, ultimately resulting in casting defects such as longitudinal cracks and concavities [8,9,10,11,12].

To design compatible mold fluxes that improve the quality of high-titanium steel ingots, numerous researchers have investigated the regulatory role of fluorides such as CaF_2_, in traditional continuous casting mold fluxes. By lowering the melting temperature, optimizing fluidity, and promoting the rational precipitation of crystalline phases, they have achieved synergistic control over lubrication and heat transfer [13,14,15,16,17]. Fluoride-containing mold fluxes are prone to fluoride volatilization at high temperatures, leading to environmental pollution, equipment corrosion, and fluctuations in slag composition [18,19,20,21]. With the increasing demands for green metallurgy and clean production, the development of fluorine-free mold fluxes has become a key direction. Therefore, the development of a fluorine-free mold flux system suitable for the continuous casting of high-titanium steel holds significant engineering and practical value. However, current research on mold fluxes for the continuous casting of high-titanium steels remains limited in several respects. First, developing fluorine-free mold fluxes that completely eliminate fluorides, suppress slag–metal interfacial reactions, and exhibit overall performance superior to that of conventional fluoride-containing slags through multicomponent synergistic regulation remains highly challenging. Second, the raw material systems used in existing studies are often poorly aligned with industrial practice. Although recent studies have made progress in B_2_O_3_-based fluorine-free mold fluxes, most have relied on chemically pure reagents. These reagents, characterized by simple compositions, low impurity levels, and minimal compositional fluctuations, are useful for elucidating fundamental mechanisms. However, they fail to reflect the compositional variability and impurity effects arising from the complex interactions among industrial-grade mineral raw materials and solid waste resources. Consequently, results obtained from chemically pure systems cannot be directly translated into industrial slag production. Moreover, the synergistic effects of multiple industrial mineral raw materials on the melting–crystallization behavior and microstructural evolution of fluorine-free mold fluxes remain insufficiently understood [22,23,24,25,26].

Based on this, this study designed a corresponding fluorine-free mold flux formulation scheme that meets the compositional and performance requirements of mold fluxes for the continuous casting of high-titanium steel. Using titanium-containing blast furnace slag as the primary base material, we selected fluoride-substituting mineral raw materials, including borax, soda ash, and monazite. We systematically investigated the mechanisms by which mineral raw materials influence the melting and crystallization behavior as well as the microstructure of fluorine-free mold flux under conditions lacking fluoride regulation. The research results provide a crucial basis for designing environmentally friendly mold fluxes suitable for the continuous casting of high-titanium steel.

## 2. Experimental Section

### 2.1. Sample Preparation

**Figure 1:**
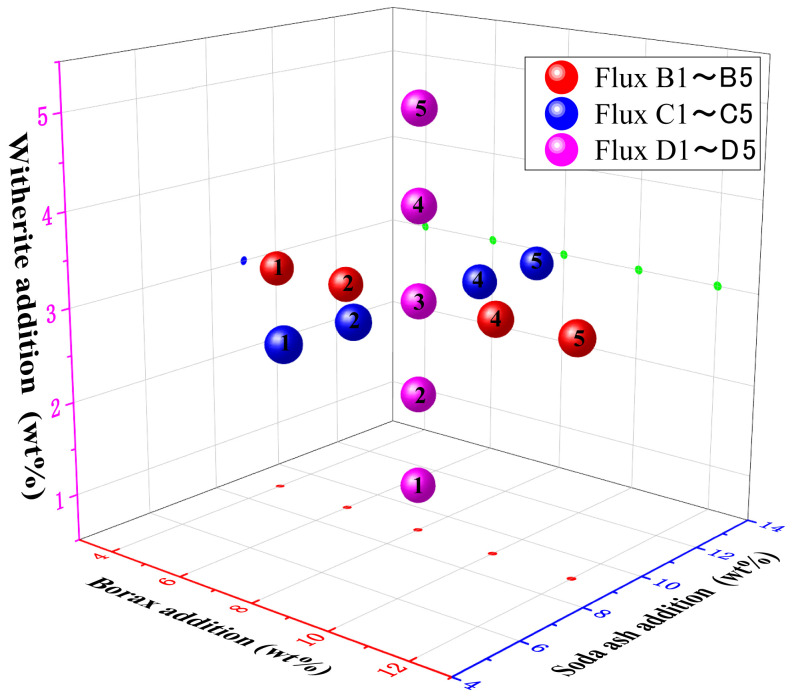
Single-factor design scheme for flux-blending experiments using mineral raw materials.

Considering the demand for low-cost mold fluxes with broad raw-material availability, as well as the service requirements of mold fluxes for continuous casting of high-titanium steel, titanium-bearing blast furnace slag, an industrial solid waste, was employed as the principal base material in this study at a proportion of 40 wt%. Limestone (28–31 wt%) and quartz sand (11–12 wt%) were used to adjust the basicity to 1.3. The contents of borax (4–12 wt%), soda ash (5–13 wt%), and witherite (1–5 wt%) were then varied independently while the contents of the other mineral raw materials were kept constant, thereby establishing single-factor flux-blending schemes with different mineral additions (Figure 1). The raw materials were sequentially mixed, melted, water-quenched, dried, and ground to obtain experimental mold flux powders with particle sizes below 0.074 mm, providing samples for subsequent testing of melting and crystallization properties. Table 1 presents the chemical compositions of the raw materials used for flux blending.

### 2.2. Methods for Testing Melting Properties

The melting characteristics of the mold fluxes were evaluated using a melting temperature tester (KFMP-1600A, Kefeng Metallurgical New Materials Co., Ltd., Luoyang, China). Prior to testing, the flux powders were sieved to 200 mesh and pressed into cylindrical specimens with a diameter and height of 3 mm. The prepared specimen was positioned on a corundum substrate at the center of the furnace chamber. During the test, the sample was heated at a programmed rate of 10 °C/min, and its shape evolution was continuously monitored by the in situ imaging system. The characteristic melting temperatures were determined from the dimensional changes in the specimen. Specifically, the temperatures corresponding to reductions in sample height to 75%, 50%, and 25% of the original height were taken as the softening temperature, hemispherical temperature, and flow temperature, respectively.

The viscosity of the mold fluxes was measured with a high-temperature rotational viscometer (RTW-13, Northeastern University, Shenyang, China). For each test, 350 g of decarburized flux was charged into a high-purity graphite crucible, heated to 1400 °C, and maintained at this temperature for 30 min to ensure complete melting and thermal homogenization. Subsequently, a pre-calibrated spindle was inserted into the molten slag. The melt was then cooled at a rate of 10 °C/min, while the viscosity was automatically recorded at selected temperature intervals after reaching steady conditions. The obtained data were used to establish viscosity–temperature relationships, and the viscosity at 1300 °C was used as a representative parameter for comparing the viscous behavior of different mold fluxes.

### 2.3. Methods for Testing Crystallization Properties

The crystallization behavior of the mold fluxes was characterized by a high-temperature in situ thermal analysis system (S/DHTT-TA-III, Chongqing University, Chongqing, China). In situ observations were carried out under prescribed isothermal temperatures and continuous cooling conditions to clarify the crystallization kinetics of the slags. The transition from a glassy melt to a crystalline structure was identified from the recorded images according to abrupt changes in surface morphology and optical transparency. Based on the crystallization onset time and temperature obtained from these observations, time-temperature-transformation (TTT) diagrams and continuous-cooling-transformation (CCT) diagrams were established. The uppermost crystallization temperature on the TTT diagram was regarded as the initial crystallization temperature, whereas the highest cooling rate at which crystallization was still detectable on the CCT diagram was defined as the critical crystallization cooling rate.

To further identify the crystalline phases and microstructural features, a fraction of the crystallized samples was ground and polished to a thin section of approximately 0.03 mm thickness. The polished section was examined with a polarizing microscope (Axio Scope A1 pol, Carl Zeiss AG, Oberkochen, Germany) to identify the crystalline phases based on their optical properties and mineralogical characteristics and to document the microstructural features. The volumetric fraction of each crystalline phase was quantified following a stereological approach. At least 20 non-overlapping, representative fields were captured, the areas of individual phases were measured with Image J 1.54p software, and according to the Delesse stereological principle, the measured area fractions were considered equivalent to the corresponding volume fractions. The total volume fraction of all crystalline phases was defined as the crystallization ratio.

In parallel, a separate portion of the crystallized samples was pulverized to a particle size below 0.074 mm and subjected to X-ray diffraction (D8 Advance, Bruker AXS, Bremen, Germany) from a 10° to 80°diffraction angle range. The resulting diffraction patterns were analyzed using the Jade 9.0 software; the interplanar spacings and relative peak intensities were extracted according to Bragg’s law and compared with standard reference databases to determine the crystalline phase assemblages.

### 2.4. Methods for Analyzing Microstructure

The structural features of the vitrified mold fluxes prepared by high-temperature water quenching were investigated using a high-resolution micro-Raman spectrometer (LabRAM HR Evolution, HORIBA, Longjumeau, France). Raman spectra were acquired with a 532 nm laser source at a power of 100 mW over a Raman shift range of 100–1600 cm^−1^, with a spectral threshold of 10 cm^−1^. Peak deconvolution was subsequently performed to quantify the distribution of Q_n_ structural units, providing insight into the microstructural evolution of the mold fluxes.

## 3. Results and Discussion

### 3.1. Effect of Mineral Raw Materials on Melting Temperature of Fluorine-Free Mold Flux

**Figure 2:**
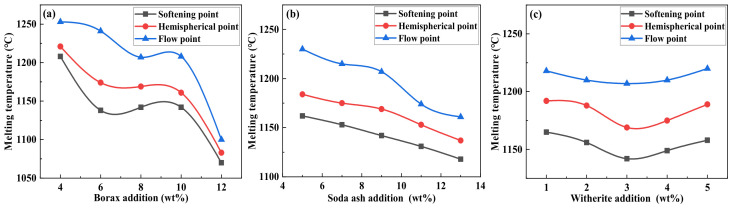
Melting temperatures of fluorine-free mold flux: (**a**) relationship between borax and melting temperature; (**b**) relationship between soda ash and melting temperature; (**c**) relationship between witherite and melting temperature.

Mold flux is a mixture without a fixed melting point. Its melting process is commonly evaluated using three characteristic temperatures: softening temperature, hemispherical temperature, and flow temperature. Based on the hemispherical point method, the temperature at which the molten flux becomes hemispherical is regarded as its melting point [27,28]. In this work, the high-temperature melting behavior of fluorine-free mold flux samples was observed in situ using a melting-point measurement apparatus. The softening, hemispherical, and flow temperatures were obtained accordingly, as illustrated in Figure 2. Fluorine-free mold fluxes with different mineral raw material contents exhibit distinct melting temperatures and melting temperature ranges. As the borax content increases, the melting temperature exhibits a segmented decline: a decrease, a plateau, and another decrease. When the borax content is 6–10 wt%, it remains in the plateau zone, with a melting point of approximately 1170 °C. At 10–12 wt% borax, excess Na_2_O (network modifier) and B_2_O_3_ (low-melting oxide) further depolymerize the silicate network, reduce liquidus temperature, and promote low-melting eutectics, causing a sharp decrease in the melting temperature. The melting temperature range of the mold flux first widens and then narrows, increasing from 45 °C to 103 °C and then decreasing to 30 °C. As the soda ash content increases, the melting temperature shows a linear, gradual decrease, dropping from 1184 °C to 1137 °C, while he melting temperature range is reduced from 68 °C to 43 °C. Compared with borax and soda ash, increasing the witherite content results in significantly smaller overall changes in melting temperature and melting temperature range; at 3 wt% witherite, the lowest melting temperature is 1169 °C.

### 3.2. Effect of Mineral Raw Materials on the Viscosity of Fluorine-Free Mold Flux

**Figure 3:**
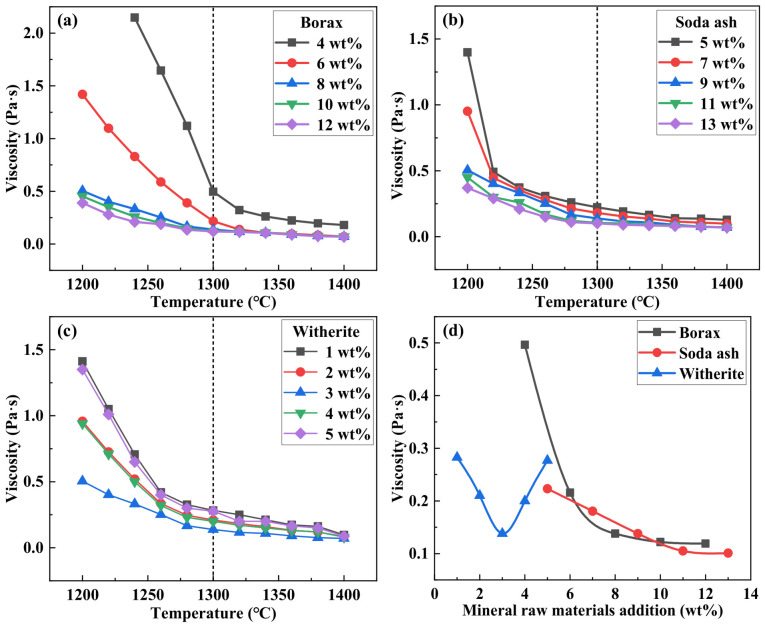
Viscosity properties of fluorine-free mold flux: (**a**–**c**) viscosity–temperature curves; (**d**) relationship between mineral raw materials and viscosity.

Viscosity, which primarily depends on the degree of silicate network polymerization, is a key indicator of the fluidity and lubricating performance of mold flux, directly influencing the liquid flux behavior and the billet lubrication in the mold [29,30]. The viscosity evolution of fluorine-free mold fluxes varied markedly with mineral raw material content, as shown in Figure 3. With increasing borax content, the viscosity of the fluorine-free mold flux first decreased sharply and then stabilized. When the borax content exceeded 8 wt%, the viscosity remained at approximately 0.137 Pa·s, indicating strong fluidity of the molten flux. As the soda ash content increased from 5 wt% to 13 wt%, the viscosity of the fluorine-free mold flux decreased slightly. With increasing witherite addition, the viscosity exhibited an initial decrease followed by an increase, attaining a minimum of 0.138 Pa·s at a witherite content of 3 wt%. The influence of different mineral raw materials on the viscosity of fluorine-free mold flux varied in magnitude. Borax exhibited the strongest ability to regulate mold flux fluidity within the interfacial space between the mold and the solidified shell, followed by witherite and soda ash. This is because the introduction of borax containing Na_2_O and B_2_O_3_ facilitates the depolymerization of the silicate network structure, promoting the formation of low-melting-point liquids. Meanwhile, the absorbed steel-derived oxides dissolve more easily, reducing the flow activation energy and enhancing the mold flux fluidity.

### 3.3. Effect of Mineral Raw Materials on the Initial Crystallization Temperature of Fluorine-Free Mold Flux

**Figure 4:**
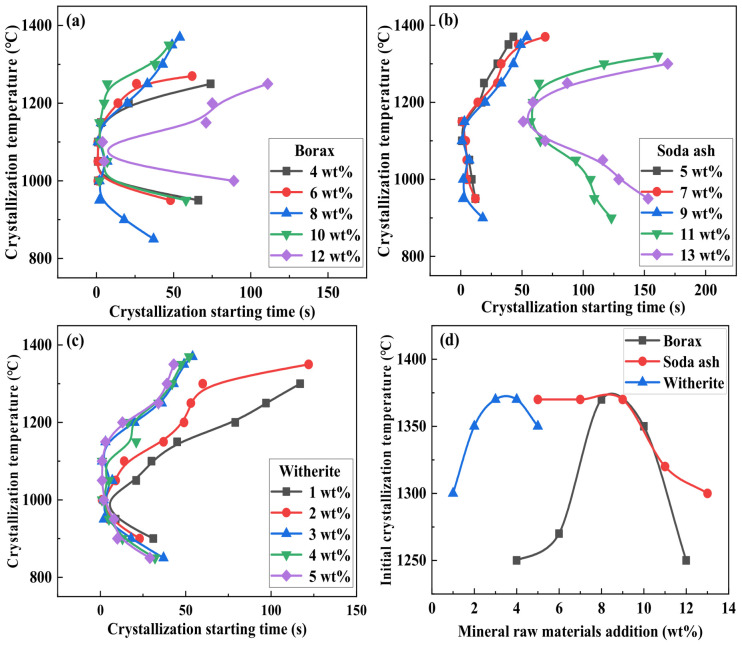
Initial crystallization temperatures of fluorine-free mold flux: (**a**–**c**) TTT curves; (**d**) relationship between mineral raw materials and initial crystallization temperature.

The TTT curves in Figure 4 show the temperature and time at which crystallization of fluorine-free mold flux begins under different target temperatures. The maximum temperature at which crystals begin to precipitate is the initial crystallization temperature of the flux and is used to characterize the nucleation ability of a fluorine-free mold flux under isothermal conditions. The results show that variations in mineral raw material content caused the initial crystallization temperature of the fluorine-free mold flux to range from 1250 °C to 1370 °C. As the temperature gradually decreased, the crystallization incubation time of the mold fluxes containing different mineral raw materials first shortened and then lengthened. With increasing borax content, the initial crystallization temperature of the fluorine-free mold flux first increased and then decreased, reaching a maximum of 1370 °C at 8 wt%. When the soda ash content increased from 5 wt% to 9 wt%, the initial crystallization temperature remained stable at 1370 °C; however, when the content exceeded 11 wt%, the initial crystallization temperature decreased, and the crystallization incubation time was significantly prolonged. Increasing the witherite content first increased and then decreased the initial crystallization temperature, while the crystallization incubation time gradually shortened. These results indicate that appropriate additions of borax and witherite enhance the nucleation ability of fluorine-free mold flux under isothermal conditions, whereas soda ash stabilizes and potentially suppresses nucleation.

### 3.4. Effect of Mineral Raw Materials on the Critical Crystallization Cooling Rate of Fluorine-Free Mold Flux

**Figure 5:**
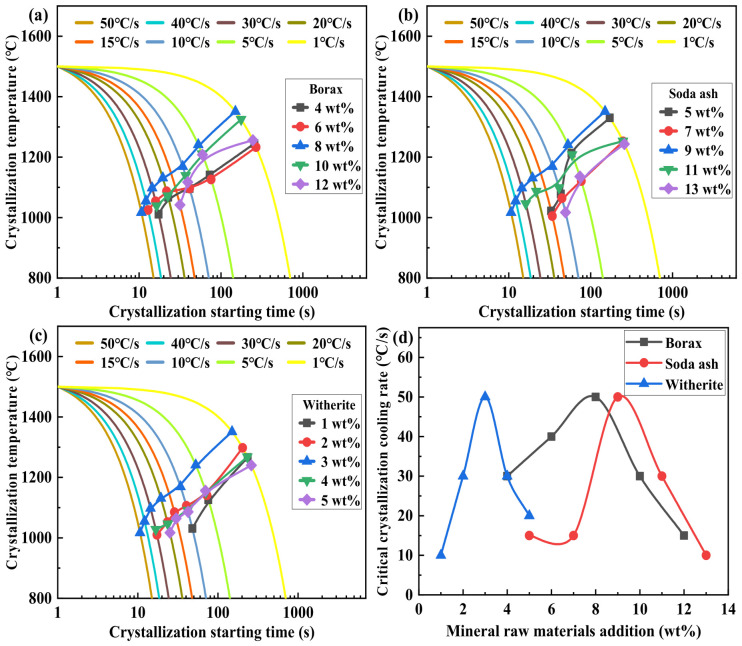
Critical crystallization cooling rate of fluorine-free mold flux: (**a**–**c**) CCT curves; (**d**) relationship between mineral raw materials and critical crystallization cooling rate.

When comparing the nucleation ability of different mold fluxes, analysis is usually conducted under defined cooling conditions. The CCT curves of fluorine-free mold fluxes, constructed from hot-thermocouple crystallization experiments and shown in Figure 5, display the temperature and time at which crystallization begins under different cooling rates. The maximum cooling rate on the CCT curve is defined as the critical crystallization cooling rate of the mold flux and is used to characterize its crystallization tendency and nucleation ability [31,32]. The CCT curves in Figure 5 indicate that changes in the content of each mineral raw material significantly affect the critical crystallization cooling rate. With increasing borax, soda ash, and witherite content, the critical cooling rate of the fluorine-free mold flux first increased and then decreased, reaching a maximum of 50 °C/s and a minimum of 10 °C/s. When the contents of borax, soda ash, and witherite were within the ranges of 6–10 wt%, 9–11 wt%, and 2–4 wt%, respectively, the critical cooling rate exceeded 30 °C/s, indicating that the fluorine-free mold flux possessed strong nucleation ability and was less sensitive to changes in cooling-rate conditions. By increasing the addition of mineral raw materials, different crystallization behaviors arise from network depolymerization and ion migration. Na_2_O/B_2_O_3_-bearing units can reduce silicate connectivity and the liquidus temperature, while BaO-bearing units provide cations for crystal nucleation. Their competition controls the crystallization tendency, crystal growth, and material performance.

### 3.5. Effect of Mineral Raw Materials on Crystalline Phases and Crystallization Ratio of Fluorine-Free Mold Flux

**Figure 6:**
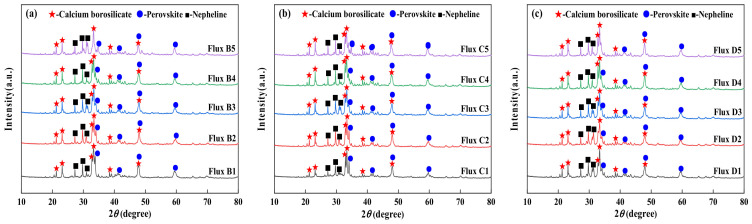
XRD patterns of crystalline phases in fluorine-free mold flux. (**a**) Flux B1~B5; (**b**) Flux C1~C5; (**c**) Flux D1~D5.

**Figure 7:**
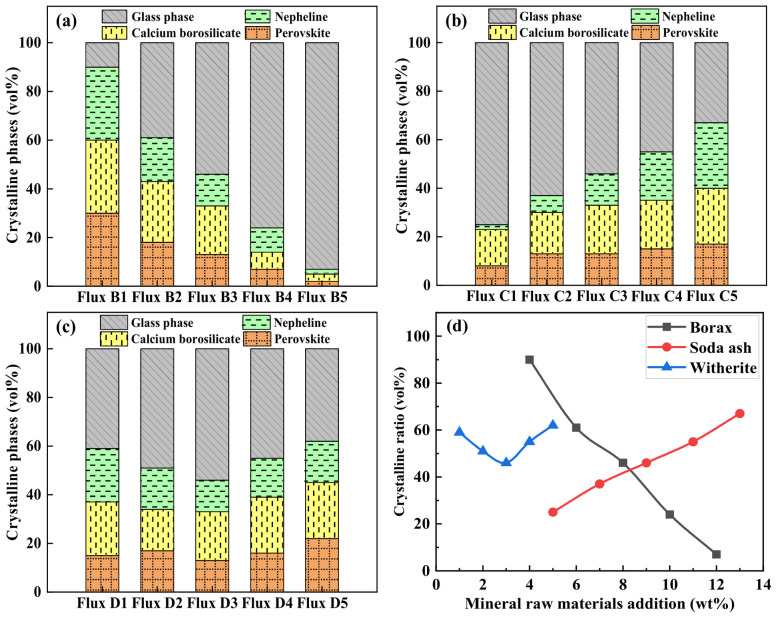
Crystalline phases and crystallization ratios of fluorine-free mold flux: (**a**–**c**) CCT curves; (**d**) relationship between mineral raw materials and crystallization ratio.

**Figure 8:**
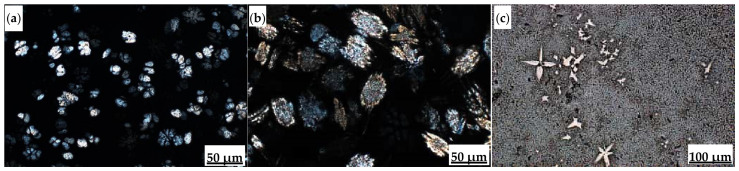

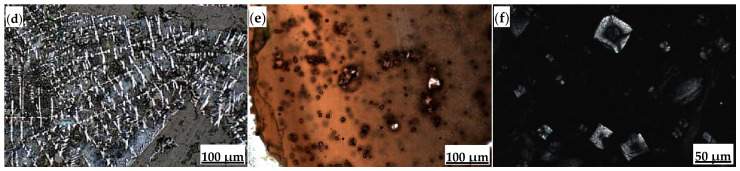
Microstructures of crystalline phases in fluorine-free mold flux: (**a**) irregular granular calcium borosilicate under transmitted cross-polarized light; (**b**) platy calcium borosilicate under transmitted cross-polarized light; (**c**) irregular granular and cross-shaped perovskite under reflected plane-polarized light; (**d**) dendritic aggregate perovskite under reflected plane-polarized light; (**e**) granular embryonic nepheline crystals under transmitted plane-polarized light; (**f**) square euhedral nepheline crystals under transmitted cross-polarized light.

During continuous casting, mold flux enters the interfacial gap between the mold and the solidifying shell. The mineralogical structure formed during solidification is also a key factor determining heat-transfer uniformity and the incidence of slab cracking [33,34,35,36]. In this study, polarizing microscopy in conjunction with X-ray diffraction was employed to identify the crystalline phase composition, phase content, crystallization ratio, and microstructural characteristics of all fluorine-free mold fluxes. The results are shown in Figure 6, Figure 7 and Figure 8.

The analysis revealed that the fluorine-free mold fluxes typically exhibited crystallization ratios in the range of 25–65 vol%, with only a few samples showing excessively high or low crystallinity. The main crystalline minerals were calcium borosilicate, nepheline, and perovskite. Calcium borosilicate mostly appeared as columnar-platy or irregular granular crystals; perovskite primarily occurred as dendritic aggregates, cross-shaped crystals, or granular crystals; and nepheline appeared as square euhedral crystals or granular embryonic crystals. Changes in mineral raw material addition significantly altered the microstructural features of the crystalline phases. Specifically, with increasing borax addition, the crystallization ratio of the mold flux decreased sharply from 90 vol% to 5 vol%, and the contents of calcium borosilicate, nepheline, and perovskite all decreased. When the borax content exceeded 6 wt%, its inhibitory effect on crystal growth and development became increasingly pronounced. This reduction results from borax depolymerizing the silicate network and suppressing crystal nucleation. The resulting lower crystallinity can improve slag lubrication and heat transfer homogeneity, reducing crack susceptibility and enhancing billet quality.

With increasing soda ash addition, the crystallization ratio increased from 25 vol% to 67 vol%, and crystal development was enhanced accordingly. In particular, nepheline precipitation increased markedly, whereas calcium borosilicate precipitation increased only slightly. When the soda ash content was lower than 11 wt%, the mold flux was more favorable for lubrication. The results indicate that soda ash-derived Na_2_O acts to depolymerize the silicate network, thereby enhancing ionic mobility and reducing the kinetic barriers for crystal nucleation and growth, ultimately leading to a pronounced promotion of crystallization. As the witherite content increased, the crystallization ratio exhibited an initial decrease followed by a subsequent increase. Compared with borax and soda ash, witherite produced much smaller variations, with the maximum difference in overall crystallization ratio being only 14 vol%. Meanwhile, the contents of crystalline mineral phases changed only slightly, indicating that crystal growth was weakly affected by witherite content and that the lubrication function of the mold flux tended to remain stable. This phenomenon can be explained by the relatively large ionic radius and limited mobility of Ba^2+^ ions released during witherite decomposition, which hinder their incorporation into the primary crystalline phase or the formation of a separate Ba-bearing phase.

### 3.6. Effect of Mineral Raw Materials on the Microstructure of Fluorine-Free Mold Flux

**Figure 9:**
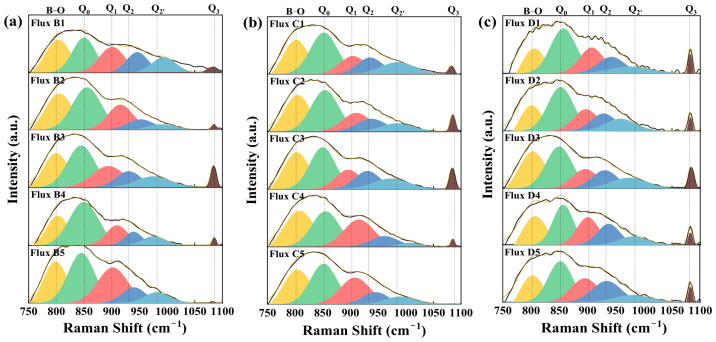
Raman spectra of the microstructure of fluorine-free mold fluxes. (**a**) Flux B1~B5; (**b**) Flux C1~C5; (**c**) Flux D1~D5.

**Figure 10:**
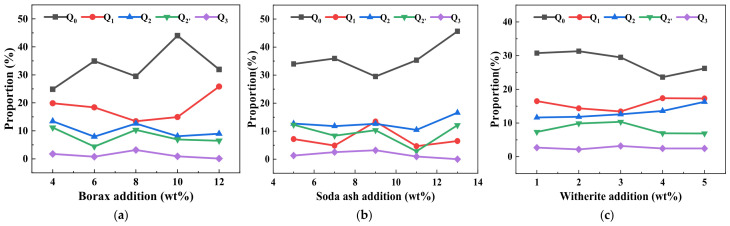
Structural units’ proportion of fluorine-free mold flux: (**a**) effect of borax content; (**b**) effect of soda ash content; (**c**) effect of witherite content.

Raman spectroscopy can characterize the distribution of structural units and chemical-bonding features in mold flux by analyzing molecular and lattice vibrations. It serves as an effective approach for assessing the polymerization degree of network structures, clarifying the influence mechanisms of network formers or modifiers on the melt structure, and elucidating the nature of crystallization phase transformations and property evolution [37,38]. The polymerization degree was evaluated from fitted Raman peak areas; Raman spectra at 750–1100 cm^−1^ were baseline-corrected, smoothed, and Gaussian-deconvoluted to identify Q_n_ silicate units; higher-Q_n_ fractions indicate stronger network polymerization. Because peak areas reflect relative structural-unit abundance, comparative Q_n_ area fractions were added to clarify mineral raw materials-induced depolymerization. Figure 9 shows the peak-deconvolution fitting results of Raman spectra in the shift range of 750–1100 cm^−1^ for fluorine-free mold fluxes containing different amounts of mineral raw materials. The relative fitted area fractions of Q_n_ structural units obtained from Raman spectra are illustrated in Figure 10.

The findings indicate that with increasing borax content, the proportion of structural units Q_0_ and Q_1_ rises progressively overall, while the proportion of highly polymerized structural units Q_2_ and Q_3_ decreases. Consequently, the overall polymerization degree of the molten flux decreases, leading to a reduced viscosity and a significant increase in ionic mobility. These changes are unfavorable for the stable arrangement of atoms and promote the vitrification tendency. Meanwhile, although the B-O structural peak area decreases, the magnitude of reduction is small, and its influence on the crystallization tendency of calcium borosilicate is correspondingly limited. As the soda ash content increases, the peak area of the B-O structural peaks in the molten flux remains largely unchanged, while their proportion shows a decreasing trend. Soda ash addition contributes to the breakdown of the network structure, leading to a shift in the structural units Q_2_ and Q_3_ toward less-polymerized Q_0_ and Q_1_. This transformation reduced melt viscosity and enhanced ion diffusion, thereby facilitating ordered atomic arrangement and strengthening the crystallization tendency of nepheline. With a gradual increase in witherite addition, the peak area of the B-O structure rises and then declines. The peak areas of structural units Q_1,_ Q_2_, and Q_3_ show no significant variation, while the proportion of the structural unit Q_0_ decreases slightly. Overall, the complexity of the molten flux structure changed little, indicating that witherite contributed, to a certain extent, to the stability of the mold flux structure and properties.

### 3.7. Optimized Design of Mold Flux for High-Titanium Steel Continuous Casting

**Figure 11:**
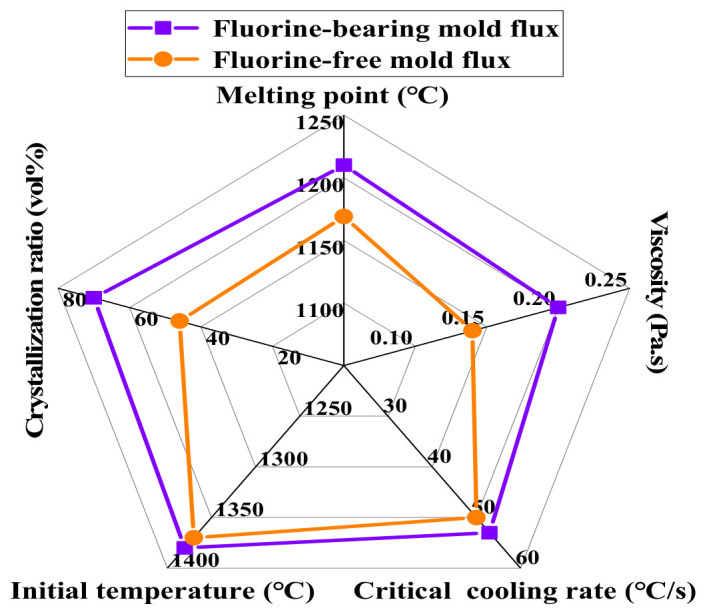
Key performance parameters between fluorine-bearing and fluorine-free mold fluxes.

**Figure 12:**
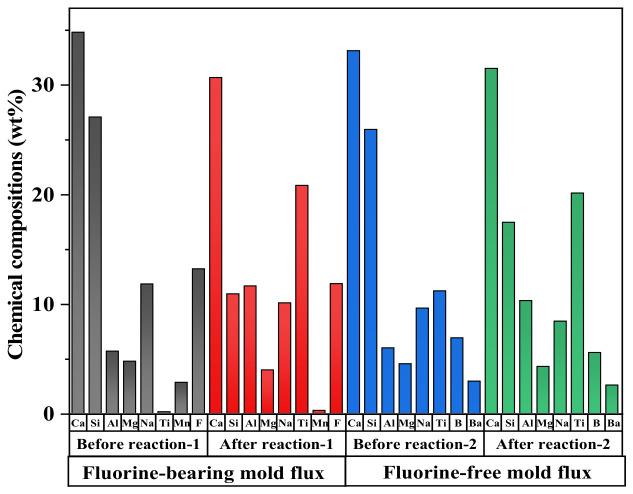
Compositional changes during slag–metal interfacial reactions between fluorine-bearing and fluorine-free mold fluxes.

Shougang Jingtang United Iron & Steel Co., Ltd., Tangshan, China, produces an ultra-high-strength high-titanium steel using a slab continuous caster. The conventional fluorine-bearing mold flux used during continuous casting presents risks of functional failure induced by slag–metal interfacial reactions, as well as fluoride pollution. In this study, an on-site investigation of high-titanium steel continuous casting was conducted. Fluorine-bearing mold flux samples and their corresponding crystallized flux film samples were collected from the production site, and the physical properties and crystalline phases of the industrial fluorine-bearing mold flux were systematically examined. On this basis, the experimental findings for the fluorine-free mold fluxes were applied to the optimized design of mold flux for high-titanium steel continuous casting. Key performance parameters, including melting point, viscosity, initial crystallization temperature, critical crystallization cooling rate, and crystallization ratio, were compared between the original fluorine-bearing flux and the optimized fluorine-free flux (Figure 11). In addition, FactSage 8.2 was used to simulate changes in composition at the slag–metal interface reactions for the two mold fluxes (Figure 12). The slag–metal interfacial reaction was simulated at 1500 °C and 1 atm using the Equilib module along with the FToxid, FTmisc, and FactPS databases. The slag-to-steel mass ratio was set as [1:100] according to the simulation design. Meanwhile, the mold flux compositions after reaction were determined by minimizing the Gibbs free energy.

The optimized fluorine-free mold flux exhibits comprehensive characteristics more suitable for high-titanium steel continuous casting, particularly for melting and crystallization regulation. Compared with the fluorine-bearing mold flux, the fluorine-free flux shows significantly lower melting point and viscosity, suggesting that it can provide superior lubrication by promoting the rapid spreading and uniform flow of liquid flux, thereby alleviating lubrication deficiency caused by slag–steel reactions during high-titanium steel continuous casting. Meanwhile, the fluorine-free flux exhibits an initial crystallization temperature and a critical crystallization cooling rate that are comparable to those of the fluorine-bearing flux. This demonstrates that, through the synergistic regulation of fluoride-substituting raw materials, the fluorine-free flux can still maintain strong crystallization tendency and heat-flux control capability. In addition, the crystallization ratio of the fluorine-free flux decreases from 70 vol% for the fluorine-bearing flux to 46 vol%, which is particularly beneficial for high-titanium steel continuous casting, because an appropriately reduced crystallization ratio can prevent nonuniform heat transfer caused by an excessively thick crystalline layer. Further analysis of compositional changes at the slag–metal interface indicates that the fluorine-bearing flux undergoes more pronounced compositional fluctuations after reacting with high-titanium steel. By contrast, the introduction of TiO_2_ into the fluorine-free flux system reduces the chemical potential difference between Ti in molten steel and the mold flux, thereby weakening the intensity of interfacial reactions and improving compositional stability during service. Overall, while maintaining appropriate crystallization properties, the optimized fluorine-free mold flux achieves lower melting point, lower viscosity, lower crystallization ratio, milder slag–steel interfacial reactions, and improved environmental friendliness. The comprehensive performance of the developed flux is comparable to that of conventional fluorine-bearing fluxes in heat-transfer control, while it offers superior lubrication, a lower crystallization ratio, and effective fluoride elimination. These advantages ensure stable continuous casting and improve the surface quality of high-titanium steel slabs.

## 4. Conclusions

This study systematically reveals the influence of borax, soda ash, and witherite on the melting–crystallization properties and microstructure of fluorine-free mold fluxes for high-titanium steel continuous casting, and provides a scientific basis for designing environmentally friendly mold fluxes by identifying an optimized composition with enhanced lubrication, heat-transfer control, and interfacial stability. The main conclusions are as follows:

(1) With increasing borax addition from 4 wt% to 12 wt%, the melting temperature and viscosity of the fluorine-free mold flux decreased in a segmented manner and the crystallization ratio sharply dropped from 90% to 5%, which was related to the depolymerization of the silicate network and the inhibition of crystal nucleation by the B_2_O_3_ and Na_2_O from borax.
(2) With increasing soda ash addition from 5 wt% to 13 wt%, the melting temperature decreased linearly and the crystallization ratio markedly increased with enhanced nepheline precipitation, which was related to the transformation of highly polymerized Q_2_ and Q_3_ units into simpler Q_1_ and Q_0_ units that facilitated ordered atomic arrangement.
(3) With increasing witherite addition from 1 wt% to 5 wt%, the melting temperature, viscosity and crystallization ratio showed relatively moderate variations, and the minimum values were achieved at 3 wt% addition, which was related to the slight influence of BaO on the polymerization degree of the molten flux.
(4) With the substitution of fluorine-bearing raw materials by borax, soda ash, and witherite in the fluorine-free mold flux, the initial crystallization temperature and critical crystallization cooling rate increased and then decreased, which was related to the competition between network depolymerization by Na_2_O, B_2_O_3_, and cation supply for crystal nucleation from BaO.
(5) The optimized fluorine-free mold flux exhibited better comprehensive performance than the conventional fluorine-bearing mold flux. It maintained suitable crystallization behavior while achieving lower melting point, viscosity, crystallization ratio, and slag–metal interfacial reaction, which was beneficial for stable lubrication and uniform heat transfer during high-titanium steel continuous casting.

## Figures and Tables

**Table 1:** Chemical compositions of raw materials for mold flux (wt%).

Raw Materials	CaO	SiO_2_	Al_2_O_3_	MgO	TiO_2_	Na_2_O	B_2_O_3_	CaCO_3_	Na_2_CO_3_	BaCO_3_
Blast furnace Slag	26.71	24.74	11.87	8.96	22.31					
Quartz sand		98.32								
Limestone								>98		
Borax						30.49	68.51			
Soda ash									>99	
Witherite										>99

## Data Availability

The original contributions presented in this study are included in the article. Further inquiries can be directed to the corresponding author.
